# Brucellosis Presenting with Febrile Pancytopenia: An Atypical Presentation of a Common Disease and Review of Brucellosis

**DOI:** 10.1155/2021/2067570

**Published:** 2021-01-04

**Authors:** Carina Chang, Bryce D. Beutler, Mark B. Ulanja, Chukwudum Uche, Milan Zdrnja

**Affiliations:** ^1^HCA Healthcare-Sunrise Health Graduate Medical Education, Nashville, TN, USA; ^2^University of Nevada, Reno School of Medicine, Department of Internal Medicine, Reno, NV, USA; ^3^Infectious Disease Associates, Las Vegas, NV, USA

## Abstract

Brucellosis is a febrile zoonotic disease caused by one of several species of the Gram-negative coccobacillus *Brucella*. It is endemic to the Middle East, sub-Saharan Africa, and Central America. However, cases have also been reported in the United States. Infection is most commonly transmitted via unpasteurized dairy products or through occupational exposure to livestock. The clinical presentation is highly variable; symptoms may include fever, myalgias, night sweats, weight loss, nausea, and vomiting. Less common features include orchitis, osteomyelitis, and sacroiliitis. In addition, pregnant women who contract brucellosis face a markedly increased risk of miscarriage. A presumptive diagnosis is typically established through correlation of patient history and classic laboratory findings, which include transaminitis, anemia, and leukopenia with relative lymphocytosis. Definitive diagnosis can only be established through isolation of *Brucella* species from blood or tissues. Treatment involves a prolonged course of multiple antibiotics; six weeks of combination therapy with aminoglycoside or rifampin and tetracycline represents the most common regimen. Healthy individuals who develop brucellosis have a generally favorable prognosis, as the case fatality rate is less than 2%. Nevertheless, early detection and treatment are essential to reduce the risk of long-term sequelae that may result from chronic, indolent disease.

## 1. Introduction

Brucellosis is a common zoonotic illness endemic to the Middle East, Central America, and sub-Saharan Africa. It is caused by the Gram-negative coccobacillus *Brucella* and is typically transmitted via unpasteurized dairy products. Presenting signs and symptoms are highly variable and may include undulating fevers, chills, myalgias, nausea, vomiting, diarrhea, and headaches. Cytopenias represent the most common laboratory findings. Disease has been nearly eradicated in the United States. However, brucellosis should be considered in individuals presenting with fevers and cytopenias who report recent travel to an endemic region.

## 2. Case Report

A 63-year-old Hispanic female with a history of hypertension, diabetes mellitus, hyperlipidemia, and gastritis presented to the emergency department by ambulance complaining of a one-day history of moderate left upper quadrant abdominal pain, melanotic stools, and fever with a maximum temperature of 102°F. The patient had been evaluated at an urgent care center five days earlier with complaints of abdominal pain, nausea, vomiting, and diarrhea; at that time, she was diagnosed with gastroenteritis and discharged home with an oral rehydration solution. However, the symptoms had progressively worsened, and the development of dark stools prompted her to seek further care. The patient denied any other symptoms. She had no previous history of melena or hematochezia and denied recent travel or sick contacts. Her outpatient medications included celecoxib, omeprazole, metoprolol, metformin, and linagliptin. Past surgical history was significant for cholecystectomy and hysterectomy. The patient had never used tobacco products, alcohol, or recreational drugs.

The patient was afebrile and hemodynamically stable in the emergency department. Physical examination was unremarkable aside from mild tenderness to palpation of the left upper and lower quadrants of the abdomen. Hemoccult testing was negative. Complete blood count revealed leukopenia (white blood cell count: 2.4 K/uL), normocytic anemia (hemoglobin: 10.4 g/dL), and thrombocytopenia (platelet count: 92 K/uL). Erythrocyte sedimentation rate was prolonged (44 millimeters/hour), and C-reactive protein was elevated (7.6 milligrams/liter). Comparison to laboratory studies obtained one year earlier showed that these abnormalities were new.

The patient was admitted for further evaluation of a suspected gastrointestinal bleed. Esophagogastroduodenoscopy was performed the following day and revealed only a large hiatal hernia; colonoscopy showed diverticula and internal hemorrhoids, but no blood was visualized. Abdominal ultrasound showed splenomegaly and hepatic steatosis; the splenic index was calculated as 333 milliliters on computed tomography scan of the abdomen. No acute processes or findings suggestive of chronic liver disease were identified on either imaging study.

The patient exhibited undulating fevers of unknown origin throughout the course of her hospitalization. Hematology/oncology was consulted for evaluation of her pancytopenia. Human immunodeficiency virus antibodies were negative. Flow cytometry showed neither circulating CD34- or CD117-positive blasts nor monotypic B-cells or atypical T-cells. Computed tomography-guided bone marrow core biopsy revealed normal cellular bone marrow with progressive trilineage hematopoiesis. The patient's pancytopenia began to improve spontaneously on her sixth day of hospitalization. Hematology/oncology concluded that the initial pancytopenia was due to an underlying reactive process.

Blood cultures that had been obtained at admission grew Gram-negative cocci on the patient's seventh day of hospitalization, and our hospital microbiology laboratory sent them to an outside microbiology laboratory for identification. The cultures were identified presumptively as *Brucella* species 16 days after having been sent out and confirmed as *Brucella melitensis* another 8 days later. *Brucella* serology (IgM/IgG) returned positive as part of an expanded fever of unknown origin workup after infectious disease consultation. Further history was obtained, and it was discovered the patient had traveled to México approximately three months prior to admission, during which time she had consumed unpasteurized milk and cheese. The patient also endorsed previously unreported symptoms that had occurred over the preceding months, including disorientation, headaches, bilateral hearing loss, and complete vision loss affecting the right eye. An extensive workup that included computed tomography scan of the head, brain magnetic resonance imaging, brain magnetic resonance angiography, and electroencephalography was performed; all testing was normal.

Based on the correlation of the patient history, laboratory findings, negative imaging studies, positive *Brucella* serology, and *Brucella melitensis* on the blood culture, a diagnosis of brucellosis was established. The patient was started on empiric treatment with oral doxycycline (100 milligrams twice daily) and oral rifampin (600 milligrams daily). Fevers resolved within two days, and with clinical improvement, the patient was discharged four days after starting oral antibiotics. The patient was unable to tolerate rifampin after one week due to severe gastrointestinal disturbances, and rifampin was discontinued. She completed six weeks of oral doxycycline with gradual and complete resolution of her symptoms. Repeat laboratory studies for complete blood count, complete metabolic panel, erythrocyte sedimentation rate, and C-reactive protein were obtained two weeks after initiation of therapy and showed no abnormal values. Blood cultures were repeated two weeks after the completion of oral doxycycline therapy and were negative. The patient made a full recovery, and she was advised to avoid unpasteurized diary on future trips to México.

## 3. Discussion

Human brucellosis represents the most common zoonotic disease worldwide, with over half a million new cases diagnosed annually [1]. It is caused by one of several species of the Gram-negative coccobacillus *Brucella*. Brucellosis has been eradicated in most developed nations. However, it is still responsible for significant morbidity and mortality in the Middle East, sub-Saharan Africa, India, and Central and South America [2, 3].

The clinical presentation and laboratory findings associated with brucellosis are unique as compared to those of other bacterial pathogens. Fevers in brucellosis are undulating; symptoms tend to wax and wane as bacteria replicate in the endoplasmic reticulum of eukaryotic cells and are periodically released into the systemic circulation. Furthermore, while bacteremia is classically characterized by leukocytosis and neutrophilia with bandemia, brucellosis is unique in that it often manifests as a febrile pancytopenia.

There are four species of *Brucella* known to cause disease in humans, each of which has a different animal host. *B. melitensis* is the most common cause of human brucellosis. The principal hosts are goats, sheep, and camels [4, 5]. Other species that can cause human brucellosis include *B. abortus* (principal host: cows), *B. suis* (principal host: pigs), and *B. canis* (principal host: dogs) [4]. In endemic countries, the disease is primarily transmitted to humans through the consumption of unpasteurized dairy products and contaminated meats. In developed countries, infection most often occurs via occupational exposure, such as inhalation of infectious aerosols in microbiology laboratories [6]. However, it can also be acquired through direct skin or mucosal contact with infected livestock [6, 7].

Regions which have achieved eradication of brucellosis have done so through mass vaccination of domestic livestock, pasteurization of dairy products, and implementation of laboratory precautions to prevent occupational exposure [8]. In countries where brucellosis has been eliminated, diagnosis of human brucellosis is almost invariably associated with travel, immigration, import of contaminated food products, or exposure of laboratory personnel [9]. In our case, the patient likely acquired brucellosis in México, but did not develop symptoms until returning to the United States three to four months later.

Classic symptoms of brucellosis include night sweats, myalgia, malaise, and prolonged, undulating fevers [9, 10]. Hepatosplenomegaly represents the most common physical examination finding. However, there are no truly pathognomonic signs, as one or multiple organ systems may be involved. Indeed, central nervous system, genitourinary, gastrointestinal, and musculoskeletal manifestations are frequently observed. Cardiovascular pathologies, such as complete atrioventricular block, have also been reported [8, 11].

*Brucella* is a facultative intracellular bacterium, and therefore, weeks or months may pass between disease transmission and the development of symptoms [9]. Bacteria are engulfed by phagocytic cells and implement various tactics to evade intracellular killing processes, allowing them to multiply within the host cell and disseminate hematogenously throughout the body. There is a strong predilection for organs of the reticuloendothelial system [5, 12]; consequently, brucellosis often presents with hematologic abnormalities. In a Turkish study of 484 cases of brucellosis, authors reported anemia in 21.5% of patients, thrombocytopenia in 18.8% of patients, and leukopenia in 14.6% of patients. Pancytopenia may occur in up to 2–14% of affected individuals [5]. There are no disease-specific findings to distinguish brucellosis-induced cytopenias from noninfectious etiologies, and therefore, brucellosis is frequently misdiagnosed as a primary hematologic disease or malignancy [7]. Fortunately, hematologic manifestations typically resolve with treatment of the underlying brucellosis.

Timely and accurate diagnosis of brucellosis is often challenging due to the prolonged incubation period and variable clinical picture [9]. Isolation of *Brucella* from blood cultures represents the gold standard diagnostic test, but often requires several weeks to yield results [1, 7, 8]. Serologic studies are only slightly less sensitive and specific than cultures and can be obtained within days [8]. Fortunately, with early detection and management, the risk of serious disability or death from brucellosis is low [10]. The World Health Organization recommends therapy with doxycycline and rifampin for six weeks. Other antibiotic regimens may include streptomycin, tetracycline, trimethoprim-sulfamethoxazole, and quinolones.

Our patient presented with vague, nonspecific symptoms and pancytopenia. Furthermore, she initially denied a travel history. Brucellosis was therefore not considered initially on the differential diagnosis, as correlation of the patient's clinical history, physical examination findings, and laboratory abnormalities was most suggestive of an underlying malignancy. Infection was not considered until Gram-negative cocci were grown in preliminary blood cultures; even then, in the absence of recent travel, *Brucella* was considered unlikely. It was not until *Brucella* serology returned positive and the patient admitted to a recent visit to México that providers began to make a presumptive diagnosis of brucellosis and initiate treatment. This case illustrates the importance of obtaining a comprehensive patient history and considering a broad differential diagnosis for any patient with a vague constellation of symptoms.

## 4. Conclusion

It is critical to consider patients in a broad clinical context, as laboratory values can seldom be evaluated in a vacuum to establish a diagnosis. The heterogeneous presentation of brucellosis demonstrates the value of obtaining a complete patient history and continually probing for new information.

## Data Availability

The case data used to support the findings of this study are available from the corresponding author upon request.

## References

[1] Buzgan T., Karahocagil M. K., Irmak H. (2010). Clinical manifestations and complications in 1028 cases of brucellosis: a retrospective evaluation and review of the literature. *International Journal of Infectious Diseases*.

[2] Sabah A. A., Aly A. M., Tawab A. H., Arafa W. A (2008). Brucellosis in Egyptian female patients. *Journal of the Egyptian Society of Parasitology*.

[3] Pappas G., Papadimitriou P., Akritidis N., Christou L., Tsianos E. V. (2006). The new global map of human brucellosis. *The Lancet Infectious Diseases*.

[4] El-Koumi M. A., Afify M., Al-Zahrani S. H. (2014). A prospective study of brucellosis in children: relative frequency of pancytopenia. *Iran J Pediatr*.

[5] Uluğ M., Yaman Y., Yapici F. (2011). Clinical and laboratory features complications and treatment outcome of brucellosis in childhood and review of the literature. *The Turkish Journal of Pediatrics*.

[6] Ulug M., Yaman Y., Yapici F., Can-Ulug N., Feigin R. D., Cherry J. D., Demmler G. J., Kaplan S. L. (2004). Brucellosis. *Textbook of Pediatric Infectious Diseases*.

[7] Kaya S., Elaldi N., Deveci O., Eskazan A., Bekcibasi M., Hosoglu S. (2018). Cytopenia in adult brucellosis patients. *Indian Journal of Medical Research*.

[8] Mantur B., Amarnath S., Shinde R. (2007). Review of clinical and laboratory features of human brucellosis. *Indian Journal of Medical Microbiology*.

[9] Norman F. F., Monge-Maillo B., Chamorro-Tojeiro S., Perez-Molina J., Lopez-Velez R. (2016). Imported brucellosis: a case series and literature review. *Travel Medicine and Infectious Disease*.

[10] Gerada A., Beeching N. J. (2016). Brucellosis and travel. *Travel Medicine and Infectious Disease*.

[11] Bilici M., Demir F., Yilmazer M. M., Bozkurt F., Tuzcu V. (2016). Brucella infection associated with complete atrioventricular block. *Balkan Medical Journal*.

[12] Franco M. P., Mulder M., Gilman R. H., Smits H. L. (2007). Human brucellosis. *The Lancet Infectious Diseases*.

